# Effect of Antihelminthic Treatment on Vaccine Immunogenicity to a Seasonal Influenza Vaccine in Primary School Children in Gabon: A Randomized Placebo-Controlled Trial

**DOI:** 10.1371/journal.pntd.0003768

**Published:** 2015-06-08

**Authors:** Sina Brückner, Selidji T. Agnandji, Stefan Berberich, Emmanuel Bache, José F. Fernandes, Brunhilde Schweiger, Marguerite Massinga Loembe, Thomas Engleitner, Bertrand Lell, Benjamin Mordmüller, Ayola A. Adegnika, Maria Yazdanbakhsh, Peter G. Kremsner, Meral Esen

**Affiliations:** 1 Institute of Tropical Medicine, University of Tübingen, Tübingen, Germany; 2 Centre de Recherches Médicales de Lambaréné, Albert Schweitzer Hospital, Lambaréné, Gabon; 3 Nationales Referenzzentrum für Influenza, Robert-Koch-Institut, Berlin, Germany; 4 Leiden Medical University Center, Department of Parasitology, Leiden, The Netherlands; George Washington University, UNITED STATES

## Abstract

**Background:**

Helminth infections are a major public health problem, especially in the tropics. Infected individuals have an altered immune response with evidence that antibody response to vaccination is impaired. Hence, treatment of helminth infections before vaccination may be a simple intervention to improve vaccine immunogenicity. In the present study we investigated whether a single-dose antihelminthic treatment influences antibody responses to a seasonal influenza vaccine in primary school children living in Gabon, Central Africa.

**Methods:**

In this placebo-controlled double-blind trial conducted in Gabon the effect of a single-dose antihelminthic treatment with 400 mg albendazole versus a placebo one month prior to immunization with a seasonal influenza vaccine was investigated. Antiviral antibody titers against all three vaccine strains were assessed by haemagglutination inhibition (HI) test at baseline (Day 0; vaccination) and four weeks (Day 28) as well as 12 weeks (Day 84) following vaccination. Vaccine-specific memory B-cell response was measured at Day 0 and Day 84 by vaccine-specific Enzyme-linked Immunospot (ELISpot) assay. The trial is registered with the Pan African Clinical Trials Registry (PACTR) (PACTR201303000434188).

**Results:**

98 school children aged 6–10 years were randomly allocated to receive either antihelminthic treatment or placebo and were vaccinated one month after the treatment. The prevalence of helminths at baseline was 21%. Vaccine-specific HI titers against at least one of the three vaccine strains increased at Day 28 and Day 84 in all participants. HI titers against both influenza A strains as well as memory B-cell response were modestly higher in the antihelminthic treated group compared to the placebo group but the difference was not statistically significant. Total but not specific IgA was elevated in the antihelminthic treated group compared to the control group at Day 28.

**Conclusion:**

In our setting antihelminthic treatment had no significant effect on influenza vaccine immunogenicity. A trend towards better antiviral and vaccine immunogenicity in the antihelminthic treated group encourages studies to be conducted with alternative treatment schedules or in populations with a higher helminth burden.

## Author Summary

Helminth infections are a major health problem in the tropics and most affected are children. The parasites are able to influence the immune system from a T-helper 1 type response to a T-helper 2 type response. There is evidence that in infected individuals the immune response following vaccination is impaired. Thus pre-treatment with a single-dose of an antihelminthic treatment before vaccination could be a simple and cost-effective intervention to improve vaccine efficacy. In the present study we investigated whether a single-dose antihelminthic treatment with albendazole influences the vaccine outcome to a seasonal influenza vaccine in primary school children living in Gabon, Central Africa. We observed a trend towards a higher anti-viral antibody titer after vaccination in the pre-treated group compared to the placebo control group, albeit not statistical significant. Furthermore we detected a higher concentration of total IgA but not of vaccine-specific IgA. In conclusion, our findings show subtle effects of antihelminthic pre-treatment but are not conclusive enough to recommend a single-dose of albendazole before vaccination to improve vaccine immunogenicity but encourage to conduct further studies in endemic areas with other treatment regiments.

## Introduction

Infection with geohelminths, mainly *Ascaris (A*.*) lumbricoides*, *Trichuris (T*.*) trichiura* and hookworm, is a major public health problem affecting 20% of the world’s population, mainly for those living in Sub-Saharan Africa (SSA). As access to public health programs is widely lacking, geohelminthiasis is considered by the World Health Organization (WHO) as one of the most neglected tropical diseases with serious health, nutritional and social outcomes for the affected individuals[1–3]. Vulnerable groups are children[2] and pregnant women[3]. Chronic infection with geohelminths has an impact on health as well as on cognitive skills[4–8] and it is known that infection with helminths leads to immune response alterations. Usually, T-helper type 2 (Th2) immune responses[9–12] are predominant and a general suppression of innate and adaptive T- and B-cell responses via the activation of regulatory T-cells (Treg) and/or induction of anti-inflammatory cytokines[10,13,14] may lead to general hyporesponsiveness of the immune system[14,15].

Vaccination is one of the most effective tools to prevent infectious diseases. Nonetheless seroconversion and therefore efficacy are variable in vaccinated individuals depending on age, environment and genetic host factors[16–18]. In addition, acute and chronic infections have an influence on vaccine outcome[19,20]. The interaction of geohelminth infection and vaccination is not well investigated although immunization programs for infants are well implemented in areas where geohelminths are highly endemic. Until now it has been shown that *A*. *lumbricoides* has an impact on the immune response induced by an oral cholera vaccine[21] and that intestinal parasites influence the outcome of a Bacillus-Calmette-Guérin (BCG) vaccination[22]. There is evidence that the presence of geohelminths, especially *T*. *trichiura* negatively influences immune responses against GMZ2, a malaria vaccine candidate[23].

Past studies from Gabon showed that Gabonese school children are heavily infected with intestinal parasites (infection rates of *A*. *lumbricoides* 46%, or *T*. *trichiura* 71%) and 74% of examined children were at least positive for one of the investigated helminths[24–26]. Regular antihelminthic treatment in high-risk groups like school children is considered as an effective tool for controlling the burden of geohelminth infection but is not widely implemented in Gabon. The WHO promotes helminth control by periodic deworming once or twice a year, depending on prevalence, as a cost-effective intervention[27,28]. However regular deworming is not yet implemented in all endemic countries[29].

Antihelminthic treatment would be a cost-effective and easy tool to reduce worm burden[30] and may simultaneously increase vaccine immunogenicity.

Therefore in the present study we investigated the effect on vaccine immunogenicity of pre-treatment with a single-dose of albendazole four weeks prior to a scheduled seasonal influenza vaccination in Gabonese primary school children.

## Materials and Methods

### Trial design and setting

For this double-blinded randomized trial healthy primary school children from Lambaréné and surroundings were randomized to receive either antihelminthic treatment (albendazole 400 mg) (Micro Lab ltd, India) or placebo (Laboratories Sterop, Belgium) four weeks (Day -28) prior to vaccination with either a seasonal influenza vaccination (VAXIGRIP, Sanofi Pasteur, season 2011/2012) intra muscularly (i.m.) (n = 98), Polysaccharide Meningococcal A+C vaccine (Sanofi Pasteur) sub cutaneously (s.c.) (n = 104) or an oral cholera vaccine (Dukoral, Sanofi Pasteur) (n = 106) administered at Day 0. Vaccinations were given in three subsequent time slots. The first cohort of primary school children was vaccinated with the influenza vaccine, the second with meningococcal vaccine and the third cohort received two times the cholera vaccine.

Inclusion criteria were age from 6 to 10 years (primary school children), a signed informed consent form (ICF), good general health upon clinical examination and no acute symptoms of geohelminths infection. Furthermore the participants and their legal representative was asked if he/she will be resident in the area until the end of the study.

Exclusion criteria were the participation in another clinical trial, known contraindication to antihelminthic treatment or to the administration of one of the chosen vaccines including i.m. or s.c. administration, known immunization against the vaccine antigens, known infection with pathogens of one of the vaccine antigens in the past except for influenza (because the influenza vaccine strain composition is different each year), any confirmed or suspected immunosuppressive or immunodeficient condition resulting from disease (e.g., malignancy, HIV infection) or immunosuppressive/cytotoxic therapy as well as acute disease at the beginning of the study and before vaccination. If a child was febrile at the scheduled time of vaccination, injections were postponed until the infection was cured. If a child had a known acute or chronic disease like malaria, AIDS or tuberculosis as well as a haemoglobin level < 7 g/dl or signs of haematuria and/or proteinuria tested by urine sticks (Combur 9 test) the child was not included and referred to the Albert Schweitzer Hospital (ASH) for treatment. If menarche was reported a pregnancy test was be performed at Day -28 and Day 0 prior to antihelmintic treatment and prior to vaccination.

Here, we report results of the first part of the study, where the children were vaccinated with the seasonal influenza vaccine.

Children, who were infected with *Schistosoma* (*S*.) *haematobium*, as well as children with any other symptomatic infection were excluded from the study and treated accordingly. All parasite positive participants (including those without symptoms) received appropriate treatment after study termination.

### Immunological investigations

Blood was taken on the day of vaccine injection (Day 0), Day 28 (four weeks after vaccination) and Day 84 (12 weeks after vaccination). The primary immunological endpoint of the study was functional antibody level measured as haemagglutination inhibition (HI) testing. To assess memory B-cell response (secondary immunological endpoint) against the vaccine antigens, an Enzyme-linked Immunospot (ELISpot) assay was performed.

### Assessment of anti-influenza antibody titers

Pre- and post-vaccination samples were analyzed by a validated microtiter haemagglutination inhibition (HI) test at the German National Reference Center for Influenza (NRZ Influenza, Robert Koch Institute (RKI), Berlin) as previously described[31]. Prior to testing, each serum was treated with receptor-degrading enzyme to inactivate non-specific inhibitors at a final serum dilution of 1:10. Sera were then diluted serially two fold into microtiter plates. Each virus strain was adjusted to 4 HA units/25 μl which was verified by back titration and 25 μl of this virus suspension was added to each of the 96 wells. After incubation at room temperature (RT) for 30 min freshly prepared 0.5% turkey red blood cells (RBCs) were added, the plates were mixed, followed by a further incubation at RT for 30 min. Human sera serving as positive controls and negative controls were included on each plate. HI titers were reported as the reciprocal of the last serum dilution that contained non-agglutinated RBCs.

### Assessment of total immunoglobuline (Ig) isotypes and subclasses

Pre- and post-vaccination samples (Day 0, Day 28 and Day 84) were analyzed by using a Multiplex assay (Biorad, Germany) for detection of multiple antibodies, like IgG subclasses (IgG1; IgG2, IgG3 and IgG4), total IgE, total IgM and total IgA at baseline, Day 28 and Day 84 post vaccination. The assay was conducted according to the manufacturer’s specifications.

### Assessment of vaccine-specific IgA by enzyme-linked immunosorbant assay (ELISA)

To assess vaccine-specific IgA concentrations plates (Nunc, Germany) were coated with 0.5 μg/ml of the vaccine antigen, incubated over night at 4°C and blocked with blocking buffer (0.3% milkpowder (Roth, Germany), 0.1% Tween-20 (Sigma, Germany) and PBS (Life technologies, USA) for 1 h at RT. Samples were plated in serial dilutions for 2 h at RT. As secondary antibody a polyclonal rabbit anti-human IgA/HRP (Dako, Germany) was used. For visualization TMB one (Kem En Tec, Denmark) and H_2_SO_4_ (Merck, Germany) was used. OD was measured using a Photometer (Phomo, Anthos, Germany) at wave length 450 nm and 620 nm as reference.

### Vaccine-specific memory B-cell ELISpot

As previously described, peripheral blood mononuclear cells (PBMCs) were frozen on Day 0 and Day 84 and later used for memory B-cell ELISpot[32,33]. In brief, PBMC were separated from heparinized full blood by gradient centrifugation (Ficoll-Plaque PLUS, GE Healthcare, Sweden), counted and frozen in 90% fetal calf serum (FCS Gold PAA, Germany) and 10% DMSO (Sigma, Germany) at -150°C. Before ELISpot, cells were thawed, counted and seeded at a density of 1*10^6^ cells per ml in RPMI 1640 (Sigma, Germany), complemented with sodium pyruvate, non-essential amino acids, L-glutamine, penicillin, streptomycin (all supplemented from Life Technologies, USA) and 10% heat inactivated FCS (FCS Gold, PAA, Germany). Maturation of circulating memory B-cells into antibody-secreting cells (ASC) was performed by *in vitro* stimulation with 2.5 μg/ml CpG-2006 (TIB-MOLBIOL, Germany) and 10 ng/ml recombinant human IL-15 (R&D systems, USA) in 24 wells cell culture plates (Corning Costar) for 6 days at 37°C, 5% CO_2_ as described[34]. Following maturation, 2*10^5^ cells were serially diluted on Ag-specific 96 well plates (Millipore, Germany). The vaccine (VAXIGRIP) was coated over night at a concentration of 5μg/ml in PBS. Plates were washed and blocked for 1 hour (h) with complete medium. Meanwhile cells were washed and counted. After seeding cells were incubated at 37°C, 5% CO_2_ for 2 h allowing them to secrete antibodies. Secreted antigen (Ag)-specific antibodies were detected using biotin labeled anti-human IgG antibody (Sigma, Germany, 1:500 in PBS, 3% BSA) and ExtrAvidin peroxidase (SIGMA, Germany, 1:600 in PBS, 5% BSA). AEC substrate (SIGMA, Germany) was added for 10 min at RT. After staining the plates were dried over night. Spots were counted by the CTL ImmunoSpot (CTL, USA).

### Assessment of parasite burden

Stool samples were collected at Day -28, Day 0 and Day 84 and analyzed by using the qualitative Merthiolate-Iodine-Formaldehyde (MIF)-technique[35]. In brief, 10 ml of Merthiolate-Formaldehyde-Solution (5% Formaldehyd, 1% Glycerine (Merck, Germany)) and Lugol’s Solution (10% potassium iodine, 5% iodine (Merck, Germany)) were added to each walnut size stool sample (approx. 5 g) filtered through a metal wire, centrifuged for 5 min 1500 rounds per minute (rpm) and analyzed by microscopy at the end of the study.

Urine was examined for the presence of *S*. *haematobium* by urine filtration method[36] using 10 ml of well mixed urine passed through a filter (12 μm pore size, Millipore, Germany). The filter was transferred to a glass slide, stained with methylene blue and analyzed by microscopy.

At Day 0 and Day 84 thick blood smears were performed to assess malaria parasites retrospectively. If a child presented with fever or any other symptom suggestive of malaria, a rapid test (Paracheck Pf) was performed and the volunteer was treated with appropriate treatment.

### Sample size calculation

We assumed that a 30% difference of immune responses between antihelminthic treatment and placebo groups is clinically relevant. In a pilot trial with the malaria vaccine candidate GMZ2 we observed a difference in antibody response greater than 30% with a standard deviation of 0.5[23]. A sample size of 45 individuals (n = 45) per group is required to detect such a difference with a power of 80% at a significance level of 0.05. To allow for 15% of loss to follow-up a total of 52 (n = 52) schoolchildren per group were required. Sample size calculation was done using R v2.9.0[37].

### Randomization, data entry and statistical analysis

The randomization and data management was performed on the “Koordobas” database system (Institute for Clinical Epidemiology and applied Biometry, University of Tübingen) The medical personal treating participants were not involved in the outcome evaluation. Group differences at follow-up visits (Day 28, Day 84) were determined with a rank based ANCOVA (Analysis of Covariance) corrected for baseline titer and gender. Differences in ELISpot counts were assessed by a Wilcoxon test. Geometric mean titers were calculated according to $10^{mean\left({log}_{10}\left(1+\text{HI-titer}\right)\right)}$. A two sided alpha of 0.05 was used as significance level.

### Ethical approval

The study was approved by the Comité d’Ethique Régional Indépendant de Lambaréné and the Comité National d´Ethique pour la Recherche du Gabon. The trial is registered with PACTR (PACTR201303000434188) and the study was conducted in accordance to the Declaration of Helsinki and followed the International Conference on Harmonization (ICH) Good Clinical Practice (GCP) guidelines. The child’s parents or their legally accepted representatives provided written informed consent before study participation.

## Results

**Fig 1:**
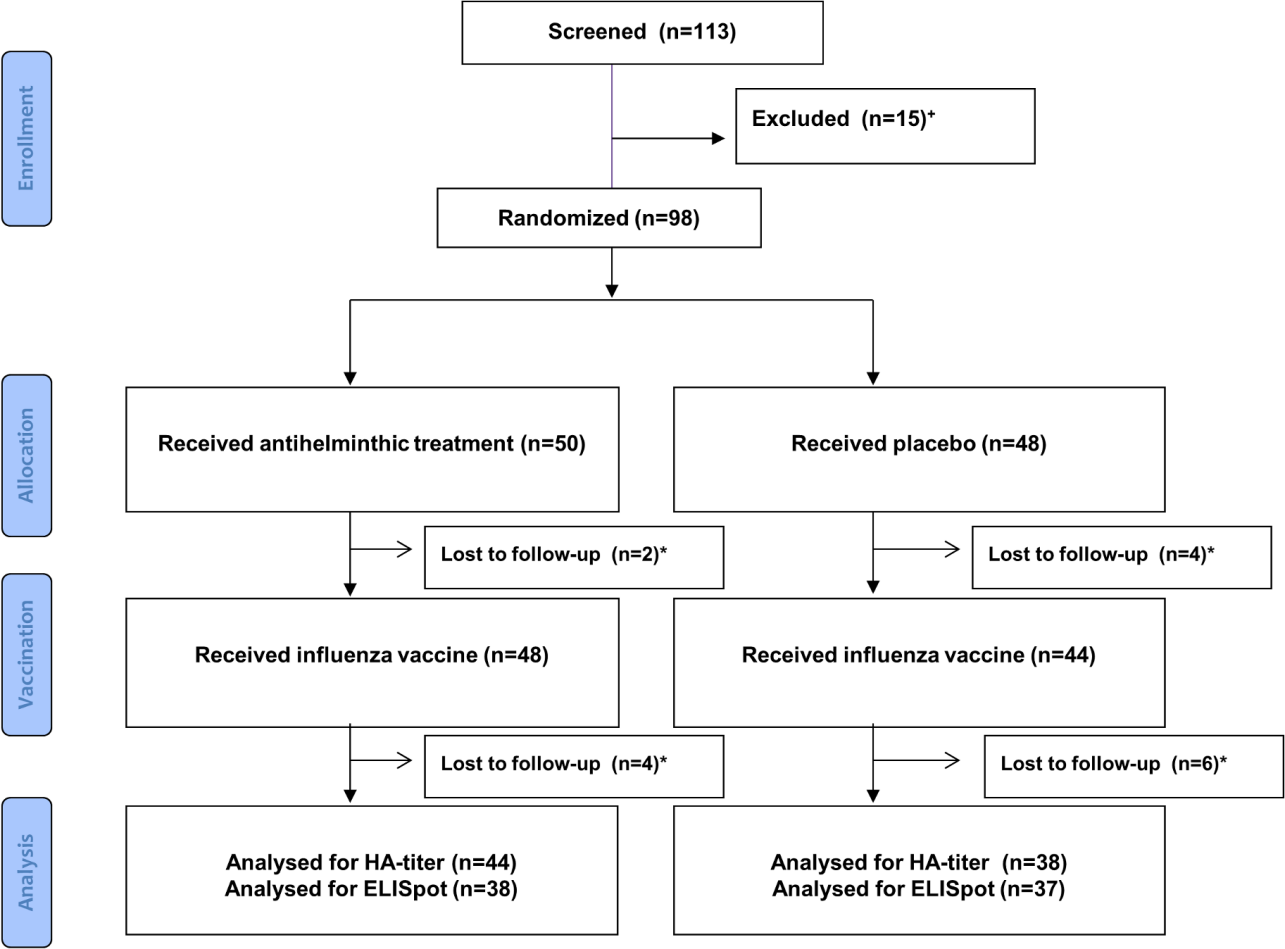
Study profile. ^+^Patients were excluded, because of infection with *S*. *haematobium*. *Participants not terminating the study are summarized as lost to follow up (n = 16).

From December 2011 until September 2012 out of 113 screened primary school children from Lambaréné and surroundings 98 were randomized to receive either antihelminthic treatment (n = 50) or placebo (n = 48). 92 participants were vaccinated with a seasonal influenza vaccine and 82 terminated the study with visit 4 at Day 84 post vaccination (Fig 1). Baseline characteristics were similar between the two groups except that the fraction of females was higher in the antihelminthic treated group (57% versus 34% in the placebo group) (Table 1). A possible effect of gender was taken into account in exploratory and sensitivity analyses.

**Table 1:** Baseline characteristics and helminth infection at day -28.

	Antihelminthic treatment	Placebo
**Gender**		
Male	22 (22.4%)	32 (32.7%)
Female	28 (29.6%)	16 (16.3%)
**Age**		
6 years	15 (15.3%)	16 (16.3%)
7 years	9 (9.2%)	8 (8.2%)
8 years	14 (14.3%)	12 (12.2%)
9 years	10 (10.2%)	12 (12.2%)
10 years	2 (2%)	0
Mean age	7.43	
**Helminth**		
Single infection		
*A*. *lumbricoides*	2 (2%)	2 (2%)
*F*. *hepatica*	0	2 (2%)
*T*. *trichiura*	4 (4%)	3 (3%)
Multiple infection		
*T*. *trichiura/ A*. *lumbricoides*	2 (2%)	4 (4%)
*T*. *trichiura/ A*. *lumbricoides/ A*. *duodenale*	0	1 (1%)
Negative	41 (41.84%)	33 (33.3%)
NA	1 (1%)	3 (3%)

### Anti-influenza antibody titers

**Fig 2:**
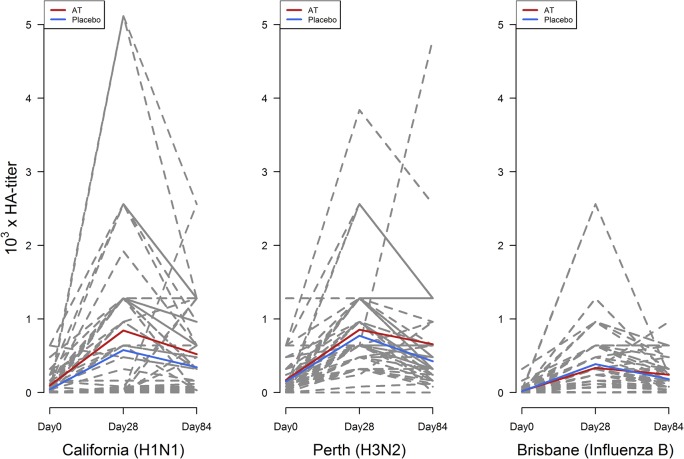
Antibody titers against the three vaccine strains at baseline (day 0), day 28 and day 84. Red lines indicate the mean of all volunteers of the antihelminthic treated group (AT) and blue lines indicate the mean of all participants of the placebo group. Dashed lines indicate antibody titers of each participant.

**Fig 3:**
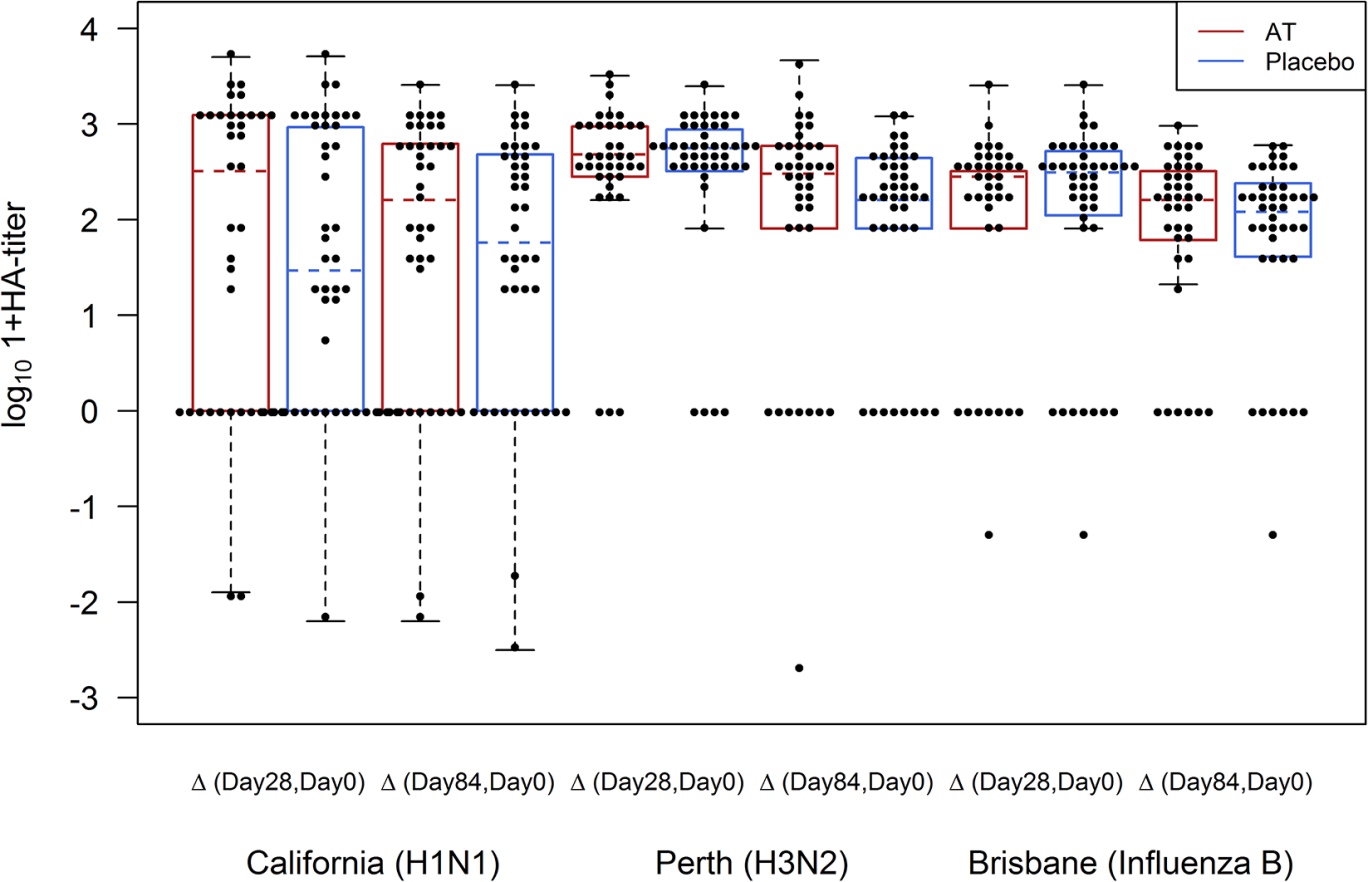
Differences of HI titers between the respective visits (day 28, day 84) and day 0 (baseline). Red and blue colors represent the pre-treated (AT) and control group.

Antibodies against the three influenza vaccine strains A/California/7/09 (A(H1N1)pdm09), A/Perth/16/09 (A(H3N2)) and B/Brisbane/60/08 were determined by HI testing. HI titers increased against at least one vaccine strain after vaccination at Day 28 and Day 84 in all participants (Fig 2). The increase of HI values for A(H1N1)pdm09 and A(H3N2) tended to be higher in the antihelminthic treated group compared to the control group but the difference was not statistically significant (Figs 2 and 3).

Thirty-four participants already had detectable antibodies at baseline for the A(H1N1)pdm09 strain. The baseline titers ranged between 15 and 640 with a median of 120 in the antihelminthic treated group and 80 in the placebo group and the GMT was 28 in the antihelminthic treated group and 15 in the control group; 19 participants in the antihelminthic treated group and 17 participants in the placebo group were without a detectable baseline HI titer. At Day 28 the HI titers increased up to 5000 (median: 640 in both groups and a GMT of 134 in the antihelminthic treated group and 84 in the control group,) and decreased until Day 84 (median: 320 in both groups and GMT of 138 in the antihelminthic treated group and 94 in the control group) (Tables 2 and 3).

**Table 2:** Median, 25% and 75% quartile of vaccine strain specific HI titers.

	Day 0	Day 28	Day 84
**A(H1N1)pdm09**			
Antihelminthic treatment	120 (0,160)	640 (0,1280)	320 (35,960)
Placebo	80 (0,60)	640 (0,960)	320 (20,480)
**A(H3N2)**			
Antihelminthic treatment	120 (20,240)	640 (320,960)	320 (280,640)
Placebo	80 (40,160)	640 (480,1280)	320 (240,600)
**Influenza B**			
Antihelminthic treatment	0 (0,40)	320 (120,320)	160 (80,320)
Placebo	0 (0,20)	320 (120,520)	160 (80,800)

**Table 3:** GMT of vaccine strain specific antibodies.

	Day 0	Day 28	Day 84
**A(H1N1)pdm09**			
Antihelminthic treatment	28	134	138
Placebo	15	84	94
**A(H3N2)**			
Antihelminthic treatment	73	554	344
Placebo	72	516	258
**Influenza B**			
Antihelminthic treatment	12	142	120
Placebo	10	164	97

Antibody titers against the strain A(H3N2) ranged from 15 to 160 (median of 120 in the antihelminthic treated group and 80 in the control group), whereas the GMT was 73 for the antihelminthic treated and 72 for the placebo group at Day 0. For this strain also an increase of antibodies at Day 28 with a median of 640 in both groups and a GMT of 554 in the antihelminthic treated group and 516 in the placebo group as well as decreasing values at Day 84 with an median of 320 in both groups and a GMT of 344 in the antihelminthic treated group and 258 in the control group were observed (Tables 2 and 3).

Antibodies against the influenza B vaccine strain had a median of 320 at Day 28 in both groups and declined until Day 84 with a median of 160 in both groups. GMT at Day 28 was 142 in the antihelminthic treated group and 164 in the placebo group and at Day 84 120 in the antihelminthic treated group and 97 in the control group. At baseline the median was 0 in both groups and the GMT was 12 for the antihelminthic treated group and 10 for the placebo group (Tables 2 and 3).

### Assessment of total Ig isotypes and subclasses

**Fig 4:**
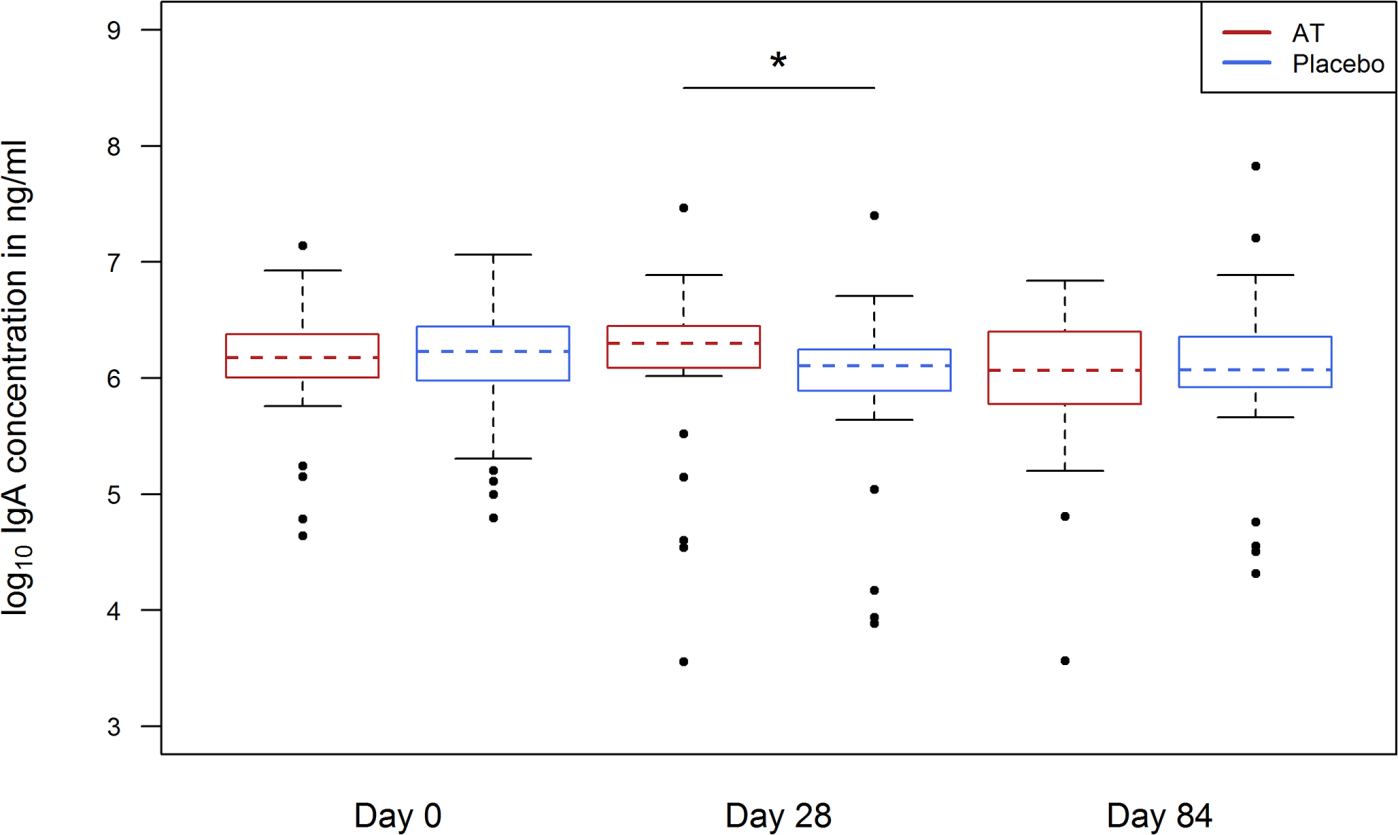
Total IgA at day 0, day 28 and day 84 in antihelminthic treated (AT) (red blots) and placebo group (blue blots).

Ig isotypes and subclasses (IgG1-4, IgA, IgE and IgM) pre- and post-vaccination were assessed by a multiplex system. Total IgA was elevated in the antihelminthic treated group compared to the control group at Day 28 (p-value 0.006) (Fig 4).

The total concentration of the subclasses IgG1 and IgG3 were slightly elevated in the antihelminthic treated group (for IgG1 p-value was 0.042 and for IgG3 p-value was 0.03 in the model-based analysis, but was not significant using the Wilcoxon-test (p-value 0.347 and 0.160)).

**Fig 5:**
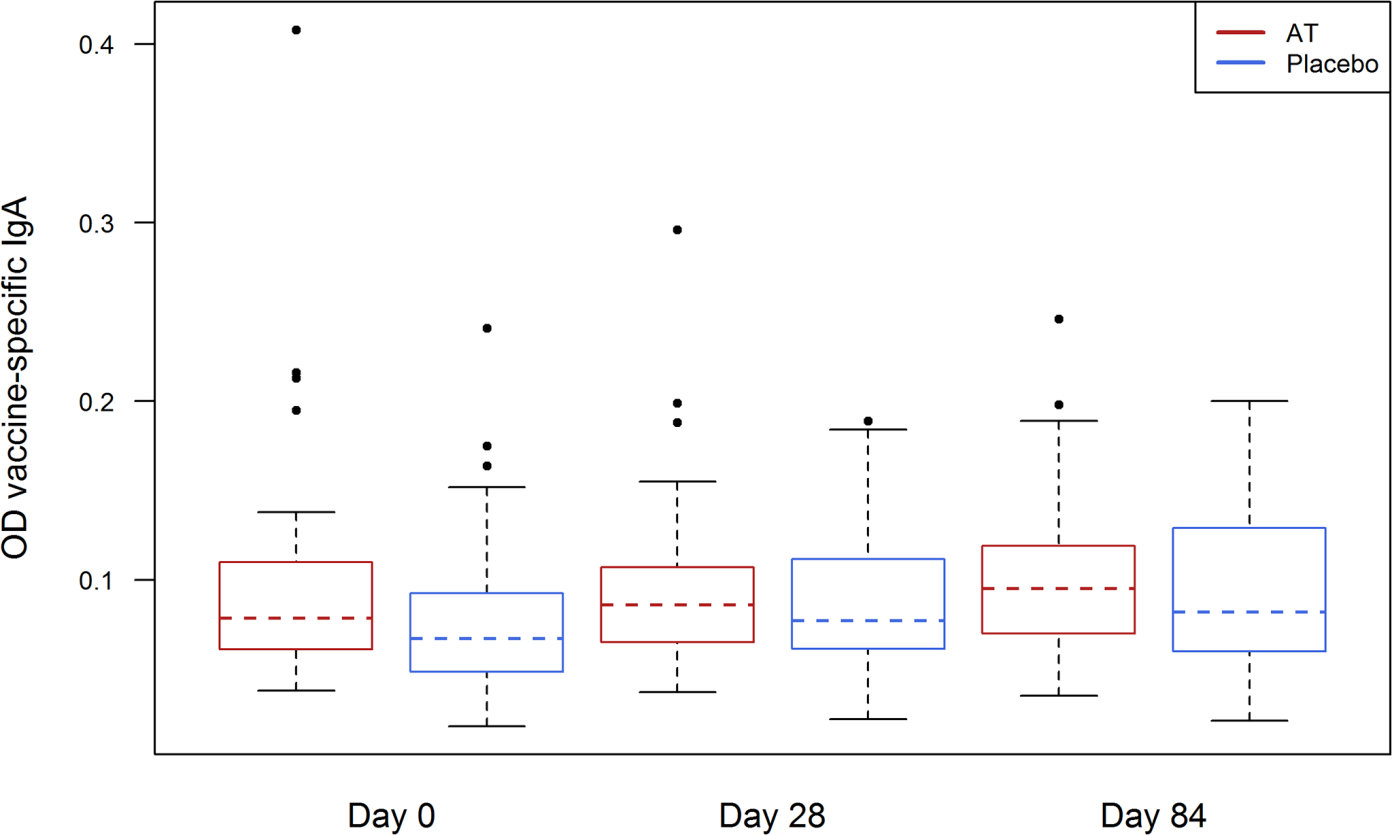
Vaccine specific IgA at day 0, day 28 and day 84 in antihelminthic treated (AT) (red) and placebo group (blue).

To evaluate whether the elevation of total IgA indicates a higher amount of vaccine-specific IgA we performed a vaccine-specific ELISA. Here, no difference of vaccine-specific IgA was detected between the antihelminthic treated and control group (Fig 5).

### Assessment of ASC

B-cell ELISpot assay to measure vaccine-specific memory B-cells was performed with samples from 75 individuals.

**Fig 6:**
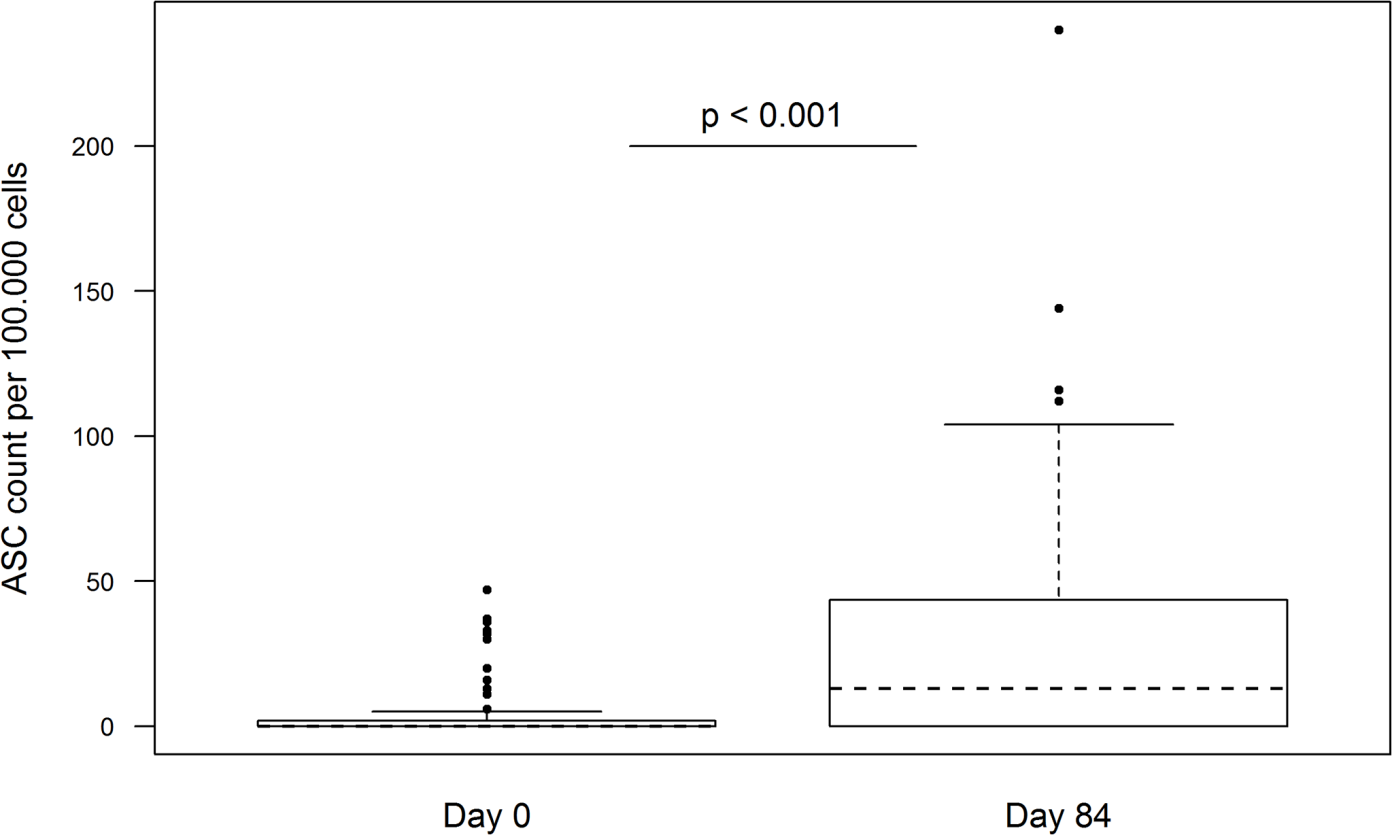
Vaccine-specific IgG ASCs determined by B-cell ELISpot at day 0 and day 84.

Antigen-specific memory B-cells were detectable in all vaccinated subjects at Day 84 (Fig 6). Median number of vaccine-specific ASC per 100,000 PBMC was 13 at Day 84 (range: 1 to 144) in all participants.

**Fig 7:**
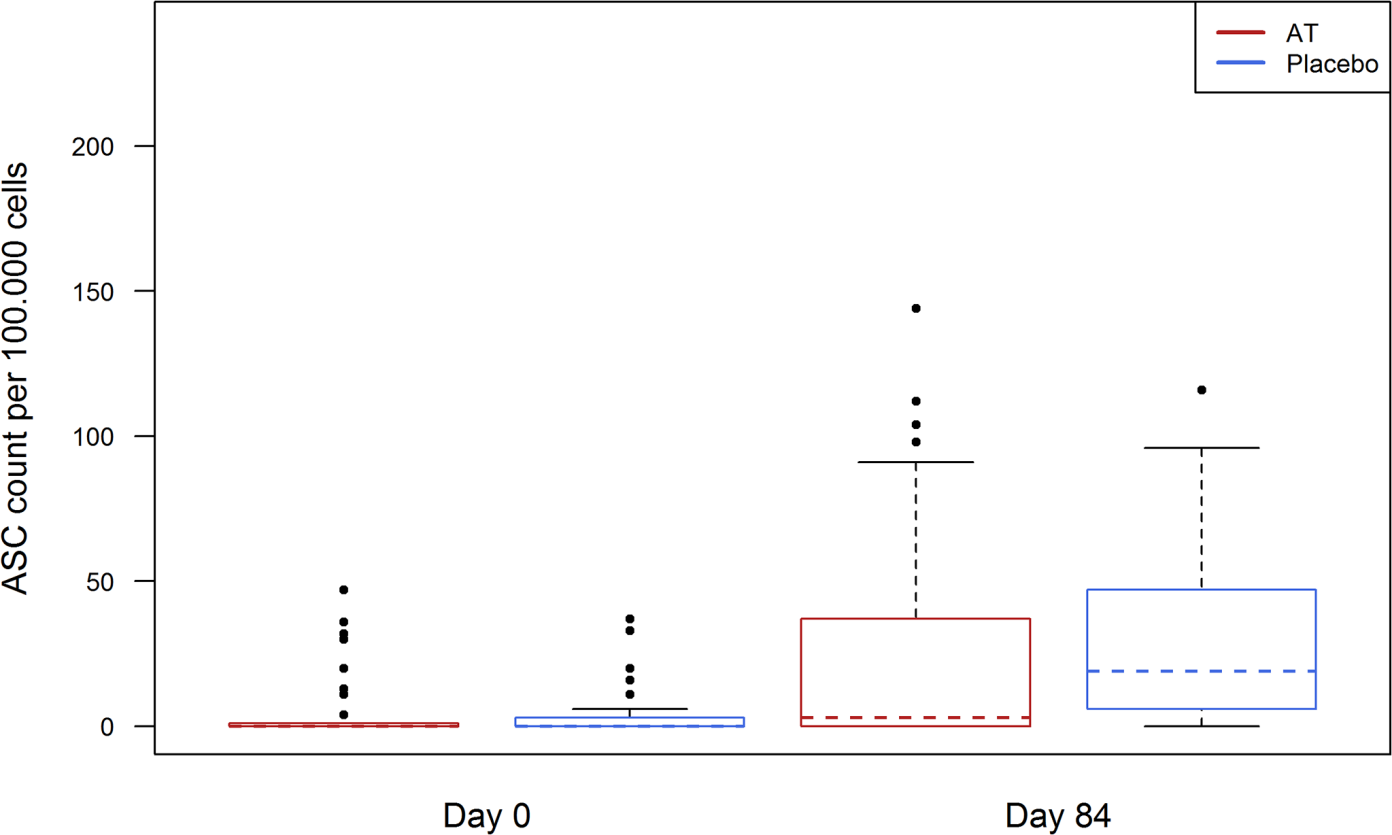
Vaccine-specific IgG ASCs at day 0 and day 84, in antihelminthic treated (AT) and placebo group. Red and blue represent antihelminthic treated (AT) and control group.

In 10 participants of the antihelminthic treated group and 12 participants of the placebo group a low number of ASCs (range: 0 to 47) was already detected at Day 0 (Fig 6). The number of detectable ASCs in the antihelminthic treated group at Day 84 ranged from 0 to 240 (median 19) and from 0 to 140 (median 3) in the placebo group (Fig 7). Nonetheless this difference was not statistically significant.

### Assessment of parasite burden

The percentage of helminth burden in our setting was 21%. From these 21%, infection with *A*. *lumbricoides* and *T*. *trichiura* was 6% for each species. The burden did not differ between the visits (Tables 1 and 4).

**Table 4:** Distribution of the worm burden in the two groups at day 0 and day 84.

	Day 0		Day 84	
	Antihelminthic treatment	Placebo	Antihelminthic treatment	Placebo
Single infection				
*A*. *lumbricoides*	2 (2.2%)	2 (2.2%)	5 (6.1%)	4 (4.9%)
*T*. *trichiura*	2 (2.2%)	3 (3.3%)	3 (3.6%)	1 (1.2%)
*S*. *haematobium*	0	0	2 (2.4%)	1 (1.2%)
*Taenia*	0	0	0	1 (1.2%)
*Tapeworm*	1 (1.1)	0	0	0
Multiple infection				
*T*. *trichiura/ A*. *lumbricoides*	1 (1.1%)	2 (2.2%)	0	3 (3.7%)
*S*. *haematobium/ T*. *trichiura*	0	0	0	1 (1.2%)
*S*. *haematobium/ A*. *lumbricoides*	0	0	1 (1.2%)	0
*T*. *trichiura/ A*. *duodenale*	1 (1.1%)	0	0	0
*T*. *trichiura/A*. *lumbricoides/ A*. *deodenale*	0	1 (1.1%)	1 (1.2%)	0
Neg	39 (42.4%)	32 (34.7%)	25 (30.5%)	24 (29.3%)
NA	2 (2.2%)	4 (4.4%)	7 (8.5%)	3 (3.7%)

At Day 0 and Day 84 9 and 10 volunteers were positive in the thick blood smear, respectively.

## Discussion

Recent studies suggest that infection with helminths influences the immunological outcome of vaccination. A study recently conducted in rural Gabon showed that children infected with helminths had an impaired antibody response against an influenza vaccine compared to those free of infection[14]. During a phase Ib trial investigating immunogenicity of the malaria vaccine candidate GMZ2 in Gabonese children those infected with *T*. *trichiura* exhibited a lower antibody response against the vaccine antigens compared to those who were not infected with the parasite[23]. Since these and other studies in animal models and humans [21,38,39] show that helminths and other intestinal infections negatively influence vaccine immunogenicity we conducted a study to test the hypothesis that antihelminthic treatment prior to vaccination will increase immune responses to vaccine antigens.

Because antibody response towards vaccine antigens is a surrogate marker for protection in areas where infectious diseases are highly endemic an effective immune response to vaccinated antigens is very important[40–42].

In the present study anti-viral antibody response was analyzed by HI test for each of the three influenza strains administered with a seasonal vaccine. The influenza vaccine was selected because a single-dose is sufficient to raise antibody responses towards a protective titer and the vaccine is not part of the Expanded Program on Immunization (EPI) in Gabon. Therefore, no or only low baseline titers would presumably be present in the study population. As expected there was an increase of HI titers against each vaccine strain after vaccination at Day 28 and Day 84 in all participants. This was observed mainly for the antibody concentration against the influenza A strains A(H1N1)pdm09 and A(H3N2). The low but frequently seen baseline HI titers against the influenza A (H1N1) and A(H3N2) in some participants suggest that these children were already in contact with circulating virus strains or that they have cross-reactive antibodies from recent circulating influenza strains or from other cross-reactive pathogens. In the present study we assessed that total IgA concentrations were higher in individuals of the antihelminthic treated group compared to the control group four weeks following vaccination. Because IgA is crucial for the control of influenza[43–45] and vaccine-specific IgA can be detected in mice following influenza vaccination[46] we also measured vaccine-specific IgA but saw no difference between the groups. Since total IgA was not different at baseline we do not know if the difference of total IgA at Day 28 is an effect of vaccination or a late effect of the antihelminthic treatment. This effect should be further investigated in more detail in particular because IgA has an important function during the defense of airborne and gastrointestinal infections. However in our setting we have not examined the role of this finding and we have not determined secretory IgA.

Besides we could show that the number of vaccine-specific ASCs representing memory B-cells was elevated in the antihelminthic treated group compared to the control group but this difference did also not reach statistical significance. We assume that the already existing ASCs at baseline in samples of some participants are due to previous influenza infections. Since the antibody titers as well as the number of ASCs were not significantly elevated in the antihelminthic treated group the question arises if a better or more effective antihelminthic treatment would have had clearer effects on the vaccine immunogenicity. The fact that the overall helminth burden did not differ between the visits implies that single-dose antihelminthic treatment is not sufficient to cure or to prevent relevant helminth infection or that reinfection occurs rapidly. This becomes more evident since Adegnika et al. very recently showed that short antihelminthic treatment regimens are efficacious to cure *A*. *lumbricoides* but not *T*. *trichiura* infection. To eliminate *T*. *trichiura* infection at least a double treatment seems to be necessary[30]. Therefore in our setting the treatment regimen was not adequate to eliminate helminths sufficiently and to reconstitute the immune system. Cooper et al. investigated the effect of a double-dose (2x200mg) albendazole treatment prior to an oral cholera vaccination leading to a highly significant increase in anti-vibriocidal antibodies[21,47]. In contrast to the present study, the investigators only included individuals carrying *A*. *lumbricoides*[21,30]. In our setting we retrospectively analyzed the helminth burden of the volunteers at the end of the study and noticed that the number of infections amongst primary school children was not as high as originally suggested from recent studies, which reduces the power of our analyses. In a study performed 2004 the overall prevalence of helminth infection was 74%[24], whereas in our study population the worm burden was only 21%. This could be due to different reasons. First, in the study conducted by van den Biggelaar et al. the Kato Katz method to detect the egg load in fresh stool samples was used[24] whereas we used the MIF technique to detect the worm burden in preserved stool samples at the end of the study to ensure all investigators were blind to the infection status of the participants. The Kato Katz method has a higher sensitivity[48] compared to the MIF-technique[49] and this may explain partially the unexpected low prevalence of helminth infection although other studies using the same methodology gave higher prevalence rates. Besides the possibility that it is a chance finding the reduction of worm burden could be due to better public health facilities and better education of the population as well as to mass drug administration (MDA) even when it is administered on an irregular basis or by uncontrolled self-mediation. Gabon is a highly endemic country for STH according to the WHO, therefore MDA is recommended but in the region where the study was performed it is not regularly administered[29]. Private use is difficult to assess since individuals do not need prescription to buy antihelminthic treatment over-the-counter[50,51] and often the individuals do not report the use of antihelminthic treatment. Furthermore it could be that a member of a household was recently treated and therefore also infection of other family members decreased[52]. Taken all this into account our in our study population the helminth burden was lower as expected for reasons which were not elicited in this study and our sample size was not powered for such a low helminth prevalence.

In conclusion, we showed that in our setting there was non-significant difference in virus-specific HI titers and ASCs against the vaccine antigens between the antihelminthic treated and the placebo group. Furthermore at Day 28 post vaccination total IgA was higher in the antihelminthic treated group compared to the control group. But there was no difference comparing vaccine-specific IgA titers. We can only speculate if a single dose antihelminthic treatment is sufficient to increase vaccine immunogenicity in a setting with higher helminth prevalence or if an appropriate more powerful treatment could contribute to a better immune response to vaccination. This has to be investigated in more detail and with different antihelminthic regiments.

## Supporting Information

S1 Checklist Consort checklist. (PDF)
