# Correction: Effect of Antihelminthic Treatment on Vaccine Immunogenicity to a Seasonal Influenza Vaccine in Primary School Children in Gabon: A Randomized Placebo-Controlled Trial

**DOI:** 10.1371/journal.pntd.0003891

**Published:** 2015-06-24

**Authors:** The PLOS Neglected Tropical Diseases Staff

There are errors in the Funding section. The correct funding information is as follows: The study was funded by the Bundesministerium für Bildung und Forschung (BMBF). The grant number is 01 KA 1009. The ministery can be found under the following url http://www.bmbf.de/.<http://www.bmbf.de/> (The author who received funding was ME.) The funders had no role in study design, data collection and analysis, decision to publish, or preparation of the manuscript." We acknowledge Deutsche Forschungsgemeinschaft and Open Access Publishing Fund of University of Tübingen for supporting publication costs.
